# Machine learning-guided green ultrasound processing enhances polyphenols, antioxidant capacity, and *in vitro* bioaccessibility of black fig vinegar

**DOI:** 10.3389/fnut.2026.1934149

**Published:** 2026-08-18

**Authors:** Burcu Çakmak Sancar, Deniz Aktaran Bala, Selinay Demirel, Remzi Gürfidan, Seydi Yıkmış, Oğuzhan Kilim, Enes Açıkgözoğlu, Mehmet Ali Şimşek, Nazan Tokatlı Demirok, Marwa Ezz El-Din Ibrahim, Nazlı Tokatlı

**Affiliations:** 1 Department of Nutrition and Dietetics, Faculty of Health Sciences, Istanbul Esenyurt University, Istanbul, Türkiye; 2 Department of Food Processing, Vocational School of Veterinary Medicine, Istanbul University-Cerrahpaşa, Istanbul, Türkiye; 3 Department of Nutrition and Dietetics, Faculty of Health Sciences, Tekirdağ Namık Kemal University, Tekirdağ, Türkiye; 4 Isparta Vocational School of Information Technologies, Isparta University of Applied Sciences, Isparta, Türkiye; 5 Department of Food Technology, Tekirdağ Namık Kemal University, Tekirdağ, Türkiye; 6 Department of Software Engineering, Faculty of Engineering and Natural Sciences, Bandırma Onyedi Eylül University, Bandırma, Türkiye; 7 Department of Food and Nutrition Sciences, College of Agricultural and Food Sciences, King Faisal University, Al-Ahsa, Saudi Arabia; 8 Department of Computer Engineering, Faculty of Engineering and Natural Sciences, Istanbul Health and Technology University, Istanbul, Türkiye

**Keywords:** bioaccessibility, black fig vinegar, functional foods, green processing, machine learning, polyphenols, ultrasound processing

## Abstract

Black fig vinegar is a functional fermented product rich in phenolic compounds and antioxidants; however, conventional thermal pasteurization may reduce its bioactive composition and functional quality. Although ultrasound processing has emerged as a promising non-thermal alternative, its optimization, predictive modeling, and influence on the *in vitro* bioaccessibility of black fig vinegar have not yet been comprehensively investigated. Therefore, this study aimed to optimize ultrasound processing conditions and evaluate their effects on phenolic composition, antioxidant capacity, and *in vitro* bioaccessibility of traditionally produced black fig vinegar using response surface methodology (RSM) integrated with ensemble machine learning algorithms. Untreated, pasteurized, and ultrasound-treated vinegars were compared, while ultrasound processing variables (processing time and amplitude) were optimized through RSM. The experimental dataset was subsequently expanded using data augmentation, and Extra Trees, CatBoost, Gradient Boosting, and AdaBoost models were developed to predict total phenolic content (TPC) and ferric reducing antioxidant power (FRAP). Among the tested algorithms, the Extra Trees model achieved the best predictive performance, with coefficients of determination (R^2^) exceeding 0.998 and prediction accuracies above 99%. Under the optimized ultrasound conditions (8 min and 58% amplitude), TPC (79.77 ± 3.69 mg GAE/100 mL), FRAP (8.87 ± 0.24 mmol TE/L), and total flavonoid content were significantly higher than those of untreated and pasteurized samples (*p* < 0.05). Ultrasound processing also significantly increased caffeic acid, catechin hydrate, chrysin, vanillin, p-coumaric acid, o-coumaric acid, and trans-ferulic acid concentrations. During simulated gastrointestinal digestion, all samples exhibited reductions in bioactive compounds; however, ultrasound-treated vinegar consistently retained higher TPC, total flavonoid content, and FRAP values, with bioaccessibility remaining approximately 30%–32%. Pearson correlation and principal component analyses further identified caffeic acid, trans-ferulic acid, rutin, and o-coumaric acid as the principal contributors to antioxidant capacity. This study integrates ultrasound optimization, RSM, data enhancement, community machine learning, phenolic profiling, and *in vitro* bioavailability assessment for black fig vinegar, presenting a robust and sustainable strategy for process optimization and the development of high-quality functional vinegar products.

## 1 Introduction

Vinegar is a fermented product produced throughout history as a preservative and food flavoring. The production process consists of two main stages: alcoholic fermentation by yeasts (*Saccharomyces cerevisiae*) and acetic acid fermentation by acetic acid bacteria (AAB) (1). Vinegars produced from different raw materials around the world vary in composition and sensory properties; grains are the primary raw material in Asia, while fruits are generally used in Western countries (2). Fruit vinegar is an abundant source of valuable bioactive molecules, including organic acids, phenolic compounds, tannins, melanoidins, tetramethylpyrazine, vitamins, and flavonoids. Fruit vinegars possess antioxidant, cardioprotective, antidiabetic, anti-obesity, anti-inflammatory, and hepatoprotective properties. These properties are essential not only as food additives but also for their health benefits. Furthermore, its effectiveness in mineral absorption, glucose regulation, and gut microbiota regulation makes vinegar a prominent choice not only as a food additive but also for its potent health benefits (3). The composition and functional properties of vinegars can vary considerably depending on the raw material used, considering these diverse production characteristics.

During vinegar fermentation, organic acids are produced, with acetic acid as the predominant compound, due to the activity of acetic acid bacteria. The presence of acetic acid is responsible for the distinctive sensory characteristics of vinegar (4). The biochemical transformations that occur during fermentation significantly influence these bioactive-rich profiles.

*Ficus carica*, commonly known as the common fig, is among the most anciently cultivated plant species, as substantiated by its significant historical legacy (5, 6). The development of value-added vinegars has seen an increased focus on selecting suitable fruit sources with rich nutritional profiles. Figs have historically been used in folk medicine to treat various ailments, particularly due to their latex content (7). Figs (*Ficus carica*) are botanically classified within the Moraceae family, a diverse taxonomic family encompassing over 800 species of trees, shrubs, and epiphytes. Members of Moraceae are predominantly cultivated in tropical and subtropical regions worldwide (8, 9). Nevertheless, nations such as Turkey, Egypt, and Algeria have emerged as leaders in the commercial promotion of *Ficus* cultivation (10). Fig fruit is characterized by its aesthetic appeal, high energy density, and substantial nutritional value (11). Given valuable nutritional characteristics, enhancing processing efficiency and preserving the bioactive potential of fig-derived products has become a key research focus.

Ultrasound is increasingly recognized as a sustainable non-thermal technology for food processing because it can reduce processing time, energy consumption, and thermal damage while preserving or enhancing quality-related compounds. Its effects are primarily associated with acoustic cavitation, involving the formation, growth, and rapid collapse of microbubbles within a liquid medium. The resulting localized shear forces, microjets, turbulence, and pressure gradients can disrupt cellular structures, enhance mass transfer, facilitate the release of intracellular compounds, and improve interactions between the food matrix and surrounding liquid. Consequently, ultrasound has been applied to the extraction of phenolic compounds, polysaccharides, pectin, pigments, and other valuable food components, as well as to hydration, microbial inactivation, fermentation, homogenization, and quality preservation processes (12–14).

Ultrasound is an environmentally friendly non-thermal technology that can improve mass transfer, disrupt cellular structures through acoustic cavitation, and enhance the extraction and preservation of bioactive compounds. Its effectiveness in food processing has been demonstrated in strawberry juice quality preservation (15), phytochemical extraction from banana inflorescence (16), AI-assisted pectin extraction from lemon peels (17), oat hydration and antioxidant enhancement (18), and enzyme-assisted extraction of bioactive polysaccharides from *Moringa oleifera* (19). These findings demonstrate that ultrasound efficiency depends strongly on the food matrix, amplitude, and treatment time, emphasizing the need for product-specific process optimization.

Despite the increasing application of ultrasound in food processing, its effects on the functional quality and gastrointestinal behavior of black fig vinegar remain insufficiently understood. To the best of our knowledge, no previous study has systematically compared untreated, thermally pasteurized, and ultrasound-treated black fig vinegar in terms of phenolic composition, antioxidant capacity, and *in vitro* bioaccessibility. Moreover, the integration of response surface methodology with machine learning to optimize ultrasound processing and predict the functional properties of black fig vinegar has not yet been investigated. Therefore, this study aimed to optimize ultrasound amplitude and treatment time, compare the bioactive and antioxidant properties of untreated, pasteurized, and ultrasound-treated vinegars, and develop ensemble machine learning models for predicting total phenolic content and FRAP values. The study also evaluated changes in individual phenolic compounds, including retention and bioaccessibility, during simulated gastrointestinal digestion. This integrated approach was intended to provide a sustainable and data-driven processing strategy for preserving the functional quality of black fig vinegar while minimizing the adverse effects associated with conventional thermal processing.

## 2 Materials and methods

### 2.1 Materials

Black fig fruits were supplied from the Aydın/Türkiye region, and unprocessed black fig vinegar was obtained by applying the traditional fermentation method (20). This vinegar obtained by Yıkmış has been subjected to three different processing methods (21). The acetic acid level of fermented black fig vinegar was determined to be approximately 4%. The processes applied are categorized as follows: the first method refers to unprocessed, traditionally produced black fig vinegar (C-BFV). The second method relates to vinegar obtained by thermal pasteurization (P-BFV). The third method (UT-BFV) refers to vinegar subjected to ultrasound treatment using conditions optimized by the reaction surface method.

### 2.2 Methods

#### 2.2.1 Dataset

**FIGURE 1:**
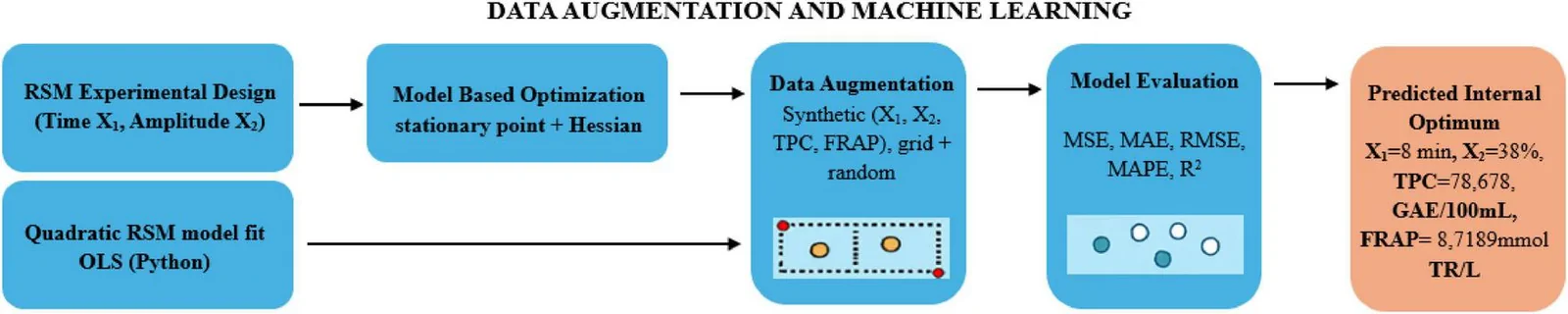
Data augmentation and machine learning. FRAP, ferric reducing antioxidant power; TPC, total phenolic content; X_1_, time; X_2_, amplitude; GAE/100 mL, gallic acid equivalent/100 mL; mmol TR/L, millimoles of Trolox equivalents per liter; OLS, Ordinary Least Squares; MSE, mean squared error; MAE, mean absolute error; RMSE, root mean squared error; MAPE, mean absolute percentage error; R^2^, coefficient of determination.

Two biochemical parameters–FRAP and TPC–were analyzed in this study across various experimental setups. The extraction time (X_1_) and amplitude (X_2_) were the independent variables responsible for these behaviors (Figure 1). The dataset was relatively small, so machine learning-based model training could not yield a specific value for the revised VSE algorithm. To fill this gap, we adopted a model-based augmentation method that enables the synthetic generation of new, realistic data points without contradicting the original experimental design. We modeled time and amplitude effects on FRAP and TPC values by fitting a second-order Response Surface Model (RSM) using Ordinary Least Squares (OLS) in Python with the statsmodels library. The regression automatically detected the relevant columns in the raw Excel data, and all numeric values were converted to floating-point format to ensure no rounding errors. Once the fitted surface was obtained, we calculated the stationary point for each model by solving the first-order partial derivative. The character of this point was determined by means of the Hessian matrix, i.e., a negative definite Hessian showed that an “enlarged domain” (local maximum) was located. While analytical optimization positioned the optimum outside experimental limits, we utilized a Differential Evolution (DE) algorithm to explore more globally within the feasible parameter bounds. The combination of analytical and evolutionary optimization ensures that the discovered optima are physically and chemically meaningful within the bounds. RSMs were used as surrogates to generate new data samples. In the first interval between X_1_ and X_2_, new input pairs were formed systematically (grid sampling) and at random (stochastic sampling). For each pair, the corresponding output (predicted response) was obtained from the regression equation. In contrast to traditional augmentation techniques that only add noise or duplicate data, this process synthesized samples based on the system’s actual relationships. That is, every new instance was chemically and experimentally consistent with actual observation. Table 1 gives metrics showing the dependence of the augmented dataset on the original dataset for TPC and FRAP values.

**TABLE 1:** Comparative similarity metrics between the TPC and FRAP datasets.

Metrics	TPC	FRAP
Range overlap X1	100.0%	100.0%
Range overlap X2	100.0%	100.0%
Convex-hull coverage (aug ⊂ orig)	45.8%	45.8%
2D hist. overlap (X1, X2) [0–1↑]	0.04	0.04
MMD^2^ (RBF, X1, X2) [0↓]	0.0402	0.0402
Sliced Wasserstein (X1, X2) [0↓]	13.9242	13.9242
Two-sample AUC (X1, X2, Y) [0.5≈]	0.637	0.569
Fréchet dist. (X1, X2, Y) [0↓]	34.9384	4.8019
Response alignment R^2^ [1↑]	1.000	0.999
Hull method	Convex hull	Convex hull

FRAP, ferric reducing antioxidant power; TPC, total phenolic content; X_1_, time; X_2_, amplitude; 2D hist. overlap, two-dimensional histogram; MMD^2^, maximum mean discrepancy squared; AUC, area under the receiver operating characteristic curve; Y, response variable; R^2^, coefficient of determination.

Similarity of comparison measurements between the TPC and FRAP datasets shows a gradual increase in both chemical measures, with minimal fluctuation in the distribution’s precision and the fit structure. The two datasets show equal overlap ranges for X_1_ (time) and X_2_ (amplitude) at 100%, confirming that the supplemented datasets were developed within the limits of the original experiments. This is to say that the augmentation process did not breach the spatial integrity of the experimental setup beyond the realized conditions. The 45.8% convex hull coverage for both datasets indicates that fewer than half of the augmented samples lie within the convex envelope of the original points. This implies that the synthetic data systematically penetrates the dense regions of the original observations, thereby increasing coverage of the design space’s outer areas. It is helpful for modeling because of the extent to which it allows for the exploration of variable interactions. The overlap of 2D histograms (0.04) and the Sliced Wasserstein distance (13.92) indicates low similarity in spatial density between the sampled and original data in both datasets. This conforms to the structured, uniform augmentation in both instances. From the perspective of a kernel, the MMD^2^ (0.0402) values are not remarkably low, indicating moderate divergence without a meaningful distributional mismatch. However, the two statistics differ in their separability and in the direction of their responses relative to the distributions. The two-sample AUCs are 0.637 for TPC and 0.569 for FRAP, indicating that the enriched TPC dataset is slightly more separable from its pristine counterpart, likely due to stronger gradients or nonlinearities in the TPC response surface. Also, TPC’s Fréchet distance (34.94) is much greater than FRAP’s (4.80), indicating greater dissimilarity in global statistical moments (covariance structure and mean). Despite these differences, there is powerful functional coherence in both datasets, as evidenced by R^2^ values of 1.000 (TPC) and 0.999 (FRAP). These results bear witness that although the geometric distributions are distinct, the augmented data preserve the underlying functional relationships between the process variables and their biochemical responses with near-perfect fidelity. The 26 rows of data in the FRAP and TPC columns were expanded to 369 rows following data augmentation. Time intervals were interpolated between 4 and 12, and amplitude values were interpolated between 40 and 80.

#### 2.2.2 Machine learning models

Table 2 presents the best hyperparameter settings identified for each machine learning model used in this research. These parameters were chosen to optimize model performance during training. For the Extra Trees and Gradient Boosting models, the depth and number of estimators were tuned to balance model complexity and generalization. The CatBoost model performed best with moderate values for depth and learning rate, ensuring convergence without overfitting. Similarly, the AdaBoost model performed best with a limited number of weak learners and a learning rate that ensured stability across both prediction tasks–TPC and FRAP.

**TABLE 2:** Best hyperparameter values for machine learning models.

Algorithms and best hyperparameters	TPC	FRAP
Extra Trees	’min_samples_split’: 2, ‘max_depth’: None, ‘n_estimators’: 100 ‘min_samples_leaf’: 1,	’min_samples_split’: 2, ‘n_estimators’: 200 ‘max_depth’: 30, ‘min_samples_leaf’: 1,
CatBoost	’learning_rate’: 0.1, ‘ depth’: 5, ‘iterations’: 200, ‘l2_leaf_reg’: 1	’learning_rate’: 0.1, ‘ depth’: 10, ‘iterations’: 200, ‘l2_leaf_reg’: 1,
Gradient Boosting	’learning_rate’: 0.1, ‘max_depth’: 5, ‘n_estimators’: 200 ‘min_samples_leaf’: 3, ‘min_samples_split’: 2,	’max_depth’: 3, ‘learning_rate’: ‘min_samples_split’: 2, 0.1,’n_estimators’: 200 ‘min_samples_leaf’: 1,
AdaBoost	’reg__estimator__max_depth’: 10, ‘reg__learning_rate’: 1.0, ‘reg__n_estimators’: 50	’reg__estimator__max_depth’: 10, ‘reg__learning_rate’: 1.0, ‘reg__n_estimators’: 200

TPC, total phenolic content; FRAP, ferric reducing antioxidant power.

#### 2.2.3 Ultrasound processing

Ultrasonic application on black fig vinegar was performed with an ultrasonic processor (Hielscher Ultrasonics, model UP200St, Berlin, Germany) using 100 mL of the vinegar sample. The device was set to operate at 200 W power output and 26 kHz frequency. During the application, amplitude levels of 40%, 55%, 70%, 85%, and 100% were evaluated, with treatment times of 2, 5, 8, 11, and 14 min. An ice water bath was used to prevent the temperature from exceeding 28 °C–32 °C during the ultrasonication process. After the process was completed, the samples were rapidly cooled in the ice bath and stored at −18 °C ± 1 °C until analysis.

#### 2.2.4 Thermal pasteurization

After the black fig vinegar samples were filled into 100 mL glass bottles, they were pasteurized for 2 min at 85 °C ± 1 °C using a water bath device (Wisd model WUC-D06H, Daihan, Wonju, Korea). After pasteurization, the samples were cooled to 20 °C ± 1 °C. These samples were defined as pasteurized black fig vinegar (P-BFV) and stored at −20 °C ± 1 °C until analytical analyses were performed.

#### 2.2.5 Determination of total phenolic compounds (TPC)

Total phenolic content (TPC) was measured using the Folin-Ciocalteau method (22). The TPC analysis was conducted by firstly mixing 2 mL of black fig vinegar with 8 mL of 80% methanol. The mixture was then centrifuged at 4000 rpm × *g* for 20 min. Assuming a dilution factor of 5 (calculated as 10/2), 50 μL of the supernatant was transferred to a glass tube. 100 μL of Folin-Ciocalteau reagent and 1500 μL of deionized water were added. After allowing the mixture to stand for 10 min, 50 μL of 20% sodium carbonate (Na_2_CO_3_) solution was added. The reaction mixture was then incubated in the dark for 2 h. Subsequently, the degree of absorption of the black fig vinegar samples was measured at 765 nm, with a blank sample used to establish a standard calibration curve. Results were expressed in milligrams of gallic acid equivalent per 100 mL of black fig vinegar samples.

#### 2.2.6 Determination of total flavonoid compounds (TFC)

The total flavonoid content (TFC) was determined by the colourimetric method as outlined by Zhishen et al. (23). A quantity of 1 ml of a diluted sample of black fig vinegar was added to each of the tubes. Following this, 4 ml of pure water and 0.3 ml of a 5% solution of sodium nitrite (NaNO_2_) were added. Following a 5-min interval, 0.3 mL of a 10% aluminum chloride (AlCl_3_) solution was added. The mixture was subjected to vigorous agitation, then allowed to repose for a further 6 min. Then, 2 mL of 1 M NaOH was added. Distilled water was added to bring the total volume to 10 mL. The samples’ absorbance was measured using a UV-Vis spectrophotometer (SP-UV/VIS-300SRB, Australia). The wavelength used was 510 nm. Analyses were performed using triple measurements and repetitions. The total flavonoid content was determined and reported in milligrams of catechin equivalent per liter (mg CE/L).

#### 2.2.7 Determination of ferric-reducing antioxidant power (FRAP)

The FRAP test was adapted for the purpose of evaluating total antioxidant activity (24). The working solution was thus formulated by combining 50 mL of acetate buffer (0.3 M, adjusted to pH 3.6), 5 mL of a 0.01 M solution of 2,4,6-tri(2-pyridyl)-1,3,5-triazine (TPTZ), and 0.02 M iron(III) chloride hexahydrate. The solution was stored at 37 °C before use. A 4.9 mL portion of the working solution was added to 0.1 mL of the test sample, and the mixture was incubated at 37 °C for 10 min. The absorbance of the resulting colored product at 593 nm was measured. In the present study, Trolox was utilized as the standard reference, and the resultant data were expressed as millimoles of Trolox equivalent (TE) per liter of black fig vinegar.

#### 2.2.8 Phenolic profile

Phenolic compounds were analyzed using reversed-phase high-performance liquid chromatography (RP-HPLC) coupled with UV-Vis detection. Samples were extracted with ethyl acetate, kept in the dark, and concentrated by solvent evaporation. Analyses were performed on a 250 mm × 4.6 mm C18 column (5 μm) at 30 °C, using a linear gradient of phosphoric acid in water and acetonitrile as the mobile phase. Sample injection volume was 10 μL, and phenolics were monitored at 280, 320, and 360 nm. Identification and quantification were performed using commercial standards, with results reported as the mean of three replicate measurements. Method validation was supported by calibration curves and bioinformatics-based data processing (25).

#### 2.2.9 Bioaccessibility determination for TPC, TFC, and FRAP values

A dialysis-based method was applied to determine the total phenolic content (TPC), total flavonoid content (TFC), and ferric reducing antioxidant capacity (FRAP) values in the bioaccessible fraction. For this procedure, a flat-type dialysis membrane (Membrane Filtration Products, Inc., Seguin, TX, USA) with a 50 kDa molecular weight cutoff was used, and the membrane was filled with 10 mL of pH 7.0 phosphate buffer. The prepared dialysis bag was immersed in the digested black fig vinegar mixture contained in a 250 mL flask. The mixture was incubated for 2 h at 37 °C under continuous stirring conditions.

After the incubation period, the dialyzable fraction (solution inside the dialysis membrane) and the non-dialyzable fraction (digested mixture remaining in the flask) were collected separately. TPC, TFC, and FRAP analyses were performed on the initial sample (pre-digestion), the post-digestion total fraction, and the dialyzable fraction. The percent bioavailability of each parameter was calculated by dividing the values measured in the dialyzable fraction by the initial concentrations in the pre-digestion black fig vinegar, and the results were reported as percentages (%). In this study, the dialyzable fraction was considered as the portion with the potential to be absorbed in the small intestine.

#### 2.2.10 Preparation of samples for *in vitro* digestion

In this study, three black fig vinegar varieties were used as model food matrices for *in vitro* digestion: control sample (C-BFV), pasteurized sample (P-BFV), and ultrasound-treated sample (UT-BFV). Samples were prepared according to the conditions specified during production and gently shaken to ensure homogeneity before analysis. For each digestion experiment, 5 mL of black fig vinegar (approximately 5 g) was measured and transferred to the digestion vessels. The digestion process, consisting of oral, gastric, and intestinal phases, was implemented based on the standard static *in vitro* digestion protocol described in the literature. All digestion stages were performed at 37 °C ± 0.3 °C to mimic the human gastrointestinal system (26).

#### 2.2.11 Oral and gastric digestion stages

In the oral phase, 5 mL of each black fig vinegar sample (C-BFV, P-BFV, UT-BFV) was mixed with 6 mL of artificial saliva solution adjusted to pH 6.8. To mimic the mechanical and enzymatic processes occurring in the oral cavity, these mixtures were incubated at 37 °C for 5 min under continuous mixing.

After the oral phase was completed, 12 mL of artificial gastric fluid prepared at pH 1.5 was added to each mixture. This phase, which reflects the conditions of the stomach environment, was performed by incubating the samples in a magnetic stirrer at 37 °C for 2 h. The suspensions obtained at the end of this process were considered complete gastric digestion samples.

#### 2.2.12 *In vitro* intestinal digestion

After completion of the gastric phase, 12 mL of artificial duodenal fluid, 6 mL of bile solution, and 2 mL of 70% bicarbonate solution were added to each sample to mimic the intestinal environment, respectively. These mixtures were incubated for 2 h at 37 °C under continuous mixing to simulate the conditions in the small intestine. The suspensions obtained at the end of this step were defined as “*in vitro* digested black fig vinegar” and used in bioaccessibility analyses.

After incubation was completed, the solution in the dialysis membrane (dialyzable fraction) and the digested sample remaining in the flask (non-dialyzable fraction) were collected separately. TPC, TFC, and FRAP analyses were performed on the initial sample (pre-digestion), the post-digestion total fraction, and the dialyzable fraction. Bioaccessibility percentages for each parameter were calculated by dividing the dialyzable fraction values by the initial concentrations before digestion, and the results were expressed as percentages (%). In this study, the dialyzable fraction was considered as the portion representing the components potentially absorbable in the small intestine.

#### 2.2.13 Statistical analysis

The experiments were repeated three times for each condition, and the results obtained are presented as mean values with standard deviation (SD). One-way analysis of variance (ANOVA) was used for statistical analysis, and the Tukey HSD (Honest Significant Difference) test was employed to determine significant differences between groups at the *p* < 0.05 level. All statistical procedures were performed using SPSS 22.0 software (SPSS Inc., Chicago, IL, USA).

## 3 Results and discussion

### 3.1 Performance findings

The predictive performances of the four ensemble-based regression models were evaluated using six statistical indicators: Mean Squared Error (MSE), Mean Absolute Error (MAE), Root Mean Squared Error (RMSE), Mean Absolute Percentage Error (MAPE), the coefficient of determination (R^2^), and overall accuracy. The obtained results are summarized in Table 3 for both biochemical responses, Total Phenolic Content (TPC) and Ferric Reducing Antioxidant Power (FRAP).

**TABLE 3:** Performance metrics for all machine learning models.

	Algorithms/ metrics	MSE	MAE	RMSE	MAPE	R^2^	Accuracy
TPC	ExtraTrees	0.1812	0.1883	0.4257	0.4236	0.99817	99.5764
	CatBoost	0.2182	0.2775	0.4672	0.5747	0.99780	99.4253
	Gradient Boosting	0.5416	0.5034	0.7359	0.9296	0.99454	99.0704
	AdaBoost	1.4684	0.9496	1.2118	1.5930	0.98521	98.4070
FRAP	ExtraTrees	0.000563	0.0130	0.0237	0.1525	0.99932	99.8475
	CatBoost	0.001214	0.0184	0.0348	0.2174	0.99853	99.7826
	Gradient Boosting	0.004243	0.0480	0.0651	0.6099	0.99485	99.3901
	AdaBoost	0.005459	0.0606	0.0739	0.7641	0.99338	99.2359

MSE, mean squared error; MAE, mean absolute error; RMSE, root mean squared error; MAPE, mean absolute percentage error; R^2^, coefficient of determination; TPC, total phenolic content; FRAP, ferric reducing antioxidant power.

In the case of TPC predictions, all algorithms achieved extremely high accuracy, with R^2^ values above 0.98, indicating a strong correlation between the predicted and experimental data. Extra Trees was the best-performing algorithm, with the lowest error (MSE = 0.1812; MAE = 0.1883) and the highest accuracy (99.58%). Its random feature selection method most likely accounted for the model’s ability to learn nonlinear relationships in the dataset after expansion. CatBoost ranked second with 99.43% accuracy and an R^2^ of 0.9978, suggesting a repetitive learning process but with a lower MAE and slightly greater MAPE, indicating local sensitivity to changes in amplitude or time. The Gradient Boosting model still maintained an acceptable level of fit (R^2^ = 0.9945), albeit with higher error metrics, indicating a deficiency in its ability to account for subtle experimental variability. On the contrary, AdaBoost had the highest errors (MSE = 1.4684; MAE = 0.9496) and a slightly reduced accuracy of 98.4%, indicating that its weak learners were insufficient to capture the response surface variability adequately.

The same trend was observed when the FRAP estimations were undertaken. All models had excellent accuracy (R^2^ > 0.99), indicating that the augmented data were highly reliable relative to the experimental values. Again, Extra Trees had the best overall performance, achieving the lowest error rates (MSE = 0.000563; RMSE = 0.0237) and an accuracy of 99.85%. The CatBoost model produced comparable results (R^2^ = 0.9985; Accuracy = 99.78%), confirming its capacity to handle non-linear relationships between process parameters and antioxidant capacity. In contrast, Gradient Boosting and AdaBoost presented slightly larger deviations, with MAPE values above 0.60 and 0.76, respectively, indicating a modest reduction in stability at extreme parameter values.

**FIGURE 2:**
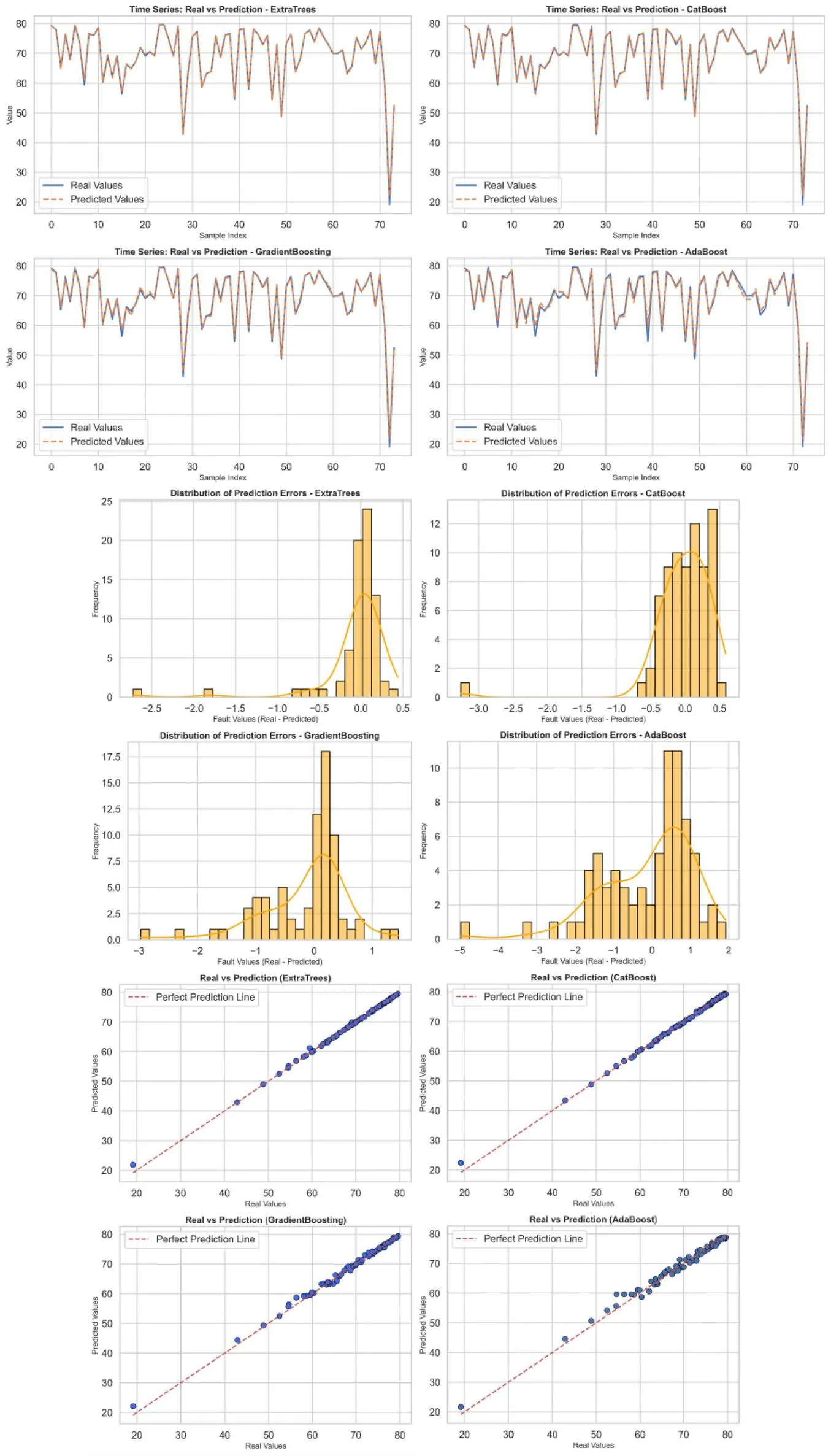
Machine learning models graphs for TPC value.

Figure 2 presents the machine learning model graphs for TPC forecasting. The orange (predicted) curves of all four models are progressing very close to the blue (actual) profile. This indicates that there is no phase error or significant lag in the sequential changes as a function of the sample index. In Extra Trees and CatBoost, the fit is particularly tight at sudden drops/peaks; in Gradient Boosting and AdaBoost, micro-scale “underfitting” is occasionally noticeable at sudden spikes. This behavior can be attributed to the boosting methods’ cumulative update mechanism being more cautious toward local extreme values. The absence of any noticeable drift throughout the series suggests that the training and validation distributions are consistent. Extra Trees and CatBoost now exhibit narrow (residual) histograms, with peak points close to zero and clearly approaching symmetry. This structure indicates weak bias, low variance, and a reasonably homogeneous conditional distribution of errors. In Gradient Boosting, the distribution is slightly more spread out; samples clustered in the right tail (positive error) imply that the model underestimates TPC in specific samples (real − predicted > 0). In AdaBoost, tails become pronounced in both directions; this suggests that stability in noisy regions may be more fragile than in other models, due to the number/depth of weak learners and the sample weight updates. The concentration of histograms around zero confirms that all models capture the underlying trend. In all models, the tight clustering of points around the red dashed line y = x indicates a strong linear fit. In particular, Extra Trees and CatBoost exhibit nearly continuous alignment along the 45° line. This shows that systematic bias is negligible and random error is low. While alignment is maintained in Gradient Boosting and AdaBoost, the distribution appears to broaden slightly, indicating minor generalization losses and occasional bias, particularly at extreme values (high TPC ranges). No significant curvature (nonlinear residual pattern) is observed in the structure of the dispersion. This indicates that the models capture the main relationships in the selected feature space.

**FIGURE 3:**
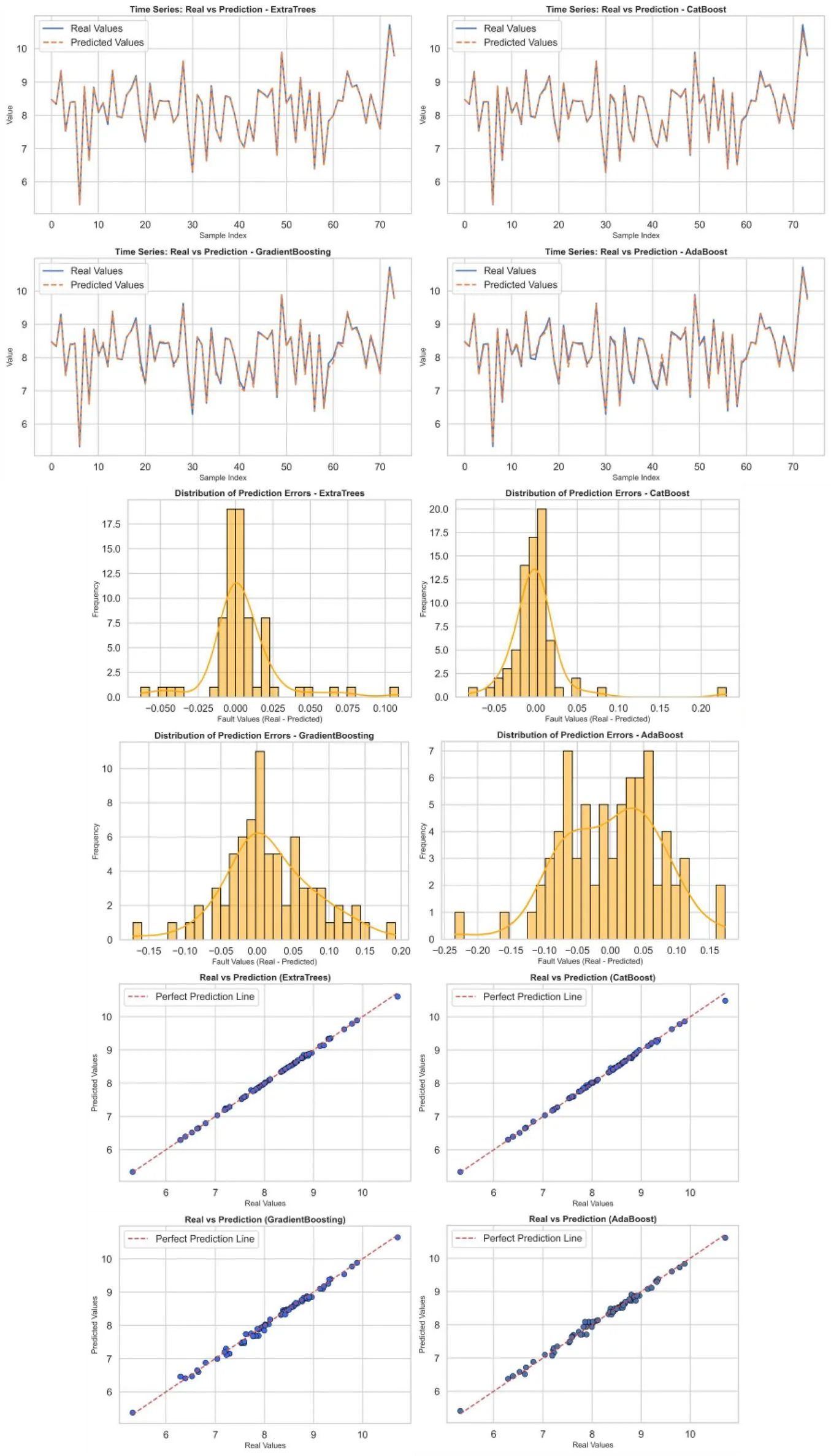
Machine learning models graphs for FRAP value.

Figure 3 shows the machine learning models for FRAP forecasting. The time-series plots show the overlap between the FRAP values predicted by each model and the experimental (real) data. For all algorithms, the blue (actual) and orange (predicted) curves are almost identical, proving that the models accurately track dynamic changes. Extra Trees and CatBoost successfully captured the data pattern, achieving high accuracy, especially during sudden dips and peaks. The occasional minor phase differences in Gradient Boosting and AdaBoost suggest that the cumulative error tends to accumulate over time, but does not translate into a measurable loss of generalization. This table shows that all models are satisfactory in terms of temporal stability, and that CatBoost does not exhibit systematic bias across successive forecasts. The histograms in the middle row show the distributions of the prediction errors (Real - Predicted) for the models. In all models, the residuals are centered around zero, indicating no bias. Extra Trees and CatBoost produced narrow, symmetric distributions, indicating that the models are low-variance and stable. In Gradient Boosting and AdaBoost, the distribution is slightly widened, suggesting that the models are more prone to volatility at extreme values (e.g., extreme FRAP measurements). The right-skewed histogram of AdaBoost suggests the model may tend to underestimate FRAP in some samples. However, the errors of all algorithms were within ±0.1, implying high prediction accuracy. The bottom row of scatter plots shows the position of each model’s predictions relative to the line of accuracy (y = x). All points are almost perfectly aligned with the red dashed line, indicating that the predictions are highly correlated with the experimental values. The Extra Trees model has the narrowest distribution and produces no systematic bias in the FRAP predictions. CatBoost performs similarly, showing a level of predictive power that can be called error-free, especially in the medium and high FRAP ranges. Although Gradient Boosting and AdaBoost graphs show minor variations around the line, their overall accuracy rates are above 99%. This linear structure shows that the FRAP data are strongly deterministic, and that all models successfully learnt this structure.

Table 4 presents a comparative analysis between the experimental and predicted values of Total Phenolic Content (TPC) and Ferric Reducing Antioxidant Power (FRAP), considering two independent variables: extraction time (X_1_) and amplitude (X_2_). The results show a very high level of agreement between model predictions and experimental data, with percentage differences of less than 1% for TPC and of a couple of thousandths for FRAP, justifying the model’s reliability and validity. The reproducibility in repeated tests under the same parameters (e.g., X_1_ = 8, X_2_ = 60) indicates high repeatability and model consistency. Parameter sensitivity analysis shows that intermediate extraction times (8–10 min) and medium amplitude levels (approximately 60%–70%) yield the highest TPC and FRAP activity, whereas extreme levels of any parameter reduce efficiency. The internally optimum condition discovered from the model (X_1_ = 8, X_2_ = 58) translates to model-predicted TPC = 78.616 mg GAE/100 mL and FRAP = 8.7139 mmol TE/L values, which are closely within the experimental plateau region, and therefore, confirm the existence of an internal optimum rather than a boundary maximum. Minor deviations in TPC estimations reflect negligible underestimation bias, whereas FRAP predictions are free of systematic error. Collectively, these findings verify the high predictive reliability, generalization capability, and experimental validity of the developed machine learning model for process optimization.

**TABLE 4:** Predictions for TPC and FRAP values.

Run no	Independent variables		Dependent variables			
	Time (X_1_)	Amplitude (X_2_)	TPC mg GAE/100 mL		FRAP (mmol TE/L)	
			Experimental data	Optimum predicted	Experimental data	Optimum predicted
1	4	60	73.24	72.76	6.85	6.85
2	8	60	78.91	78.67	8.73	8.72
3	8	60	78.91	78.67	8.84	8.72
4	8	40	68.35	67.96	8.52	8.44
5	8	60	78.91	78.67	8.85	8.72
6	10	70	60.64	60.60	9.48	9.52
7	12	60	56.03	55.76	8.80	8.70
8	8	60	78.50	78.67	8.80	8.72
9	8	80	59.73	59.88	8.97	8.88
10	6	70	78.30	77.84	7.73	7.64
11	8	60	78.12	78.67	8.64	8.72
12	8	60	78.12	78.67	8.76	8.72
13	8	40	67.67	67.96	8.43	8.44
14	8	60	78.12	78.67	8.76	8.72
15	6	50	73.56	72.93	8.42	8.35
16	8	60	78.00	78.67	8.77	8.72
17	10	50	73.26	73.45	8.30	8.36
18	4	60	72.51	72.76	6.78	6.85
19	6	70	77.51	77.84	7.65	7.64
20	6	50	72.82	72.93	8.33	8.35
21	10	70	60.03	60.60	9.52	9.52
22	12	60	55.47	55.76	8.72	8.70
23	8	60	77.72	78.67	8.72	8.72
24	8	80	59.13	59.88	8.88	8.88
25	8	60	78.26	78.67	8.85	8.72
26	10	50	73.26	73.45	8.30	8.36
Optimum	8	58	78.616		8.7139	
optimization						
parameters)						
Experimental values			79.77 ± 3.69		8.87 ± 0.24	
% difference			1.45%		1.80%	

FRAP, ferric reducing antioxidant power; TPC, total phenolic content; X_1_, Time; X_2_, amplitude; GAE/100 mL, gallic acid equivalent/100 mL; mmol TR/L, millimoles of Trolox equivalents per liter.

The findings presented in this study demonstrate that advanced ML algorithms, such as ANN and XGBoost, offer superior performance and reliability compared to traditional RSM approaches for modeling and optimizing non-linear food processing processes, such as ultrasound-assisted extraction (27). ANN’s highly adaptive and flexible fitting capability has established it as a powerful tool for modeling complex biochemical systems (27). For example, in Yıkmış et al. (28)’s jujube vinegar study, ANN demonstrated higher suitability (R^2^) and accuracy than RSM in optimizing TPC, TFC, DPPH, and CUPRAC values (28). Similarly, ANN integrated with a Genetic Algorithm (GA) has been successfully applied to optimize ultrasound-assisted pectin extraction, achieving a coefficient of determination (R^2^) of 0.99 and outperforming conventional RSM models in prediction accuracy while reducing modeling errors. The optimized conditions predicted by the ANN–GA model showed excellent agreement with experimental results, highlighting the robustness of machine learning approaches for optimizing the recovery of bioactive compounds from food matrices and improving process efficiency (29).

Ensemble learning methods such as ExtraTrees and XGBoost demonstrate high accuracy and generalization ability in process optimization and bioactive compound prediction (30).

The XGBoost algorithm stands out due to its high prediction accuracy, ability to prevent overfitting, and computational efficiency (30). In the study by Levent et al. (31), the XGBoost model made highly reliable predictions, keeping the mean relative error (MRE) below 2% for TPC, FRAP, and total chlorophyll under optimal conditions (31). Similarly, in a study predicting antioxidant capacity (TEAC) using electrochemical impedance spectroscopy (EIS) data, XGBoost outperformed simple ML models such as Multiple Linear Regression (MLR) and Random Forest Regression (RFR), recording an average deviation of less than 5% compared to traditional DPPH analyses (30).

ML algorithms are valuable not only for their predictive capabilities but also for their capacity to provide mechanical insights into the process (30, 31). XGBoost’s feature importance analysis and SHAP values enable the interpretation of the effect of critical process variables (31). In EIS biosensor modeling, the Charge Transfer Resistance (Rct) and Double Layer Capacitance (Cdl) parameters were found to be the most effective predictors, accounting for approximately 75% of the variance in antioxidant capacity predictions (30). This finding indicates that the model successfully learned the fundamental electrochemical mechanisms at the biosensor interface (30).

The generalization ability of ML models depends on the quality and size of the dataset used (31). In datasets with small sample sizes (e.g., when performing 5-fold cross-validation), it has been observed that even XGBoost cannot demonstrate consistent fit performance and yields negative R^2^ values (31). This situation indicates that the model’s complexity exceeds the capacity of the available dataset and suggests a potential risk of overfitting [Levent et al. (31)]. However, the model’s ability to predict with relative errors below 2% under optimized experimental conditions demonstrates its reliability in terms of practical applicability and biological consistency (31).

In this study, ExtraTrees stood out, while in the garden cress water-themed study, the XGBoost algorithm determined the optimal conditions by making highly reliable predictions with an average relative error (MRE) below 2% for TPC, FRAP, and chlorophyll (31). XGBoost also outperformed simple ML models such as Random Forest Regression (RFR) and Multiple Linear Regression (MLR) in predicting the antioxidant properties of fruit juices using Electrochemical Impedance Spectroscopy (EIS) data, recording an average deviation of less than 5% (30). This demonstrates that both XGBoost and ExtraTrees are superior and reliable tools for modeling complex, non-linear food data compared to traditional statistical methods.

### 3.2 Total bioactive compounds

**FIGURE 4:**
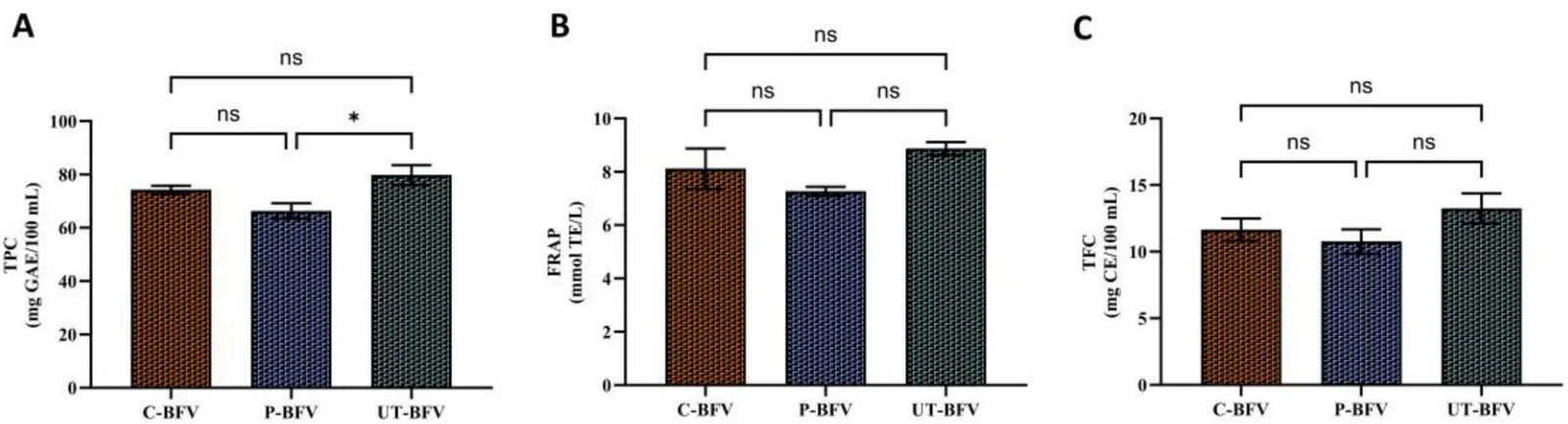
Total flavonoid content (TFC, mg CE/100 mL), ferric reducing antioxidant power (FRAP, mmol TE/L), and total phenolic content (TPC, mg GAE/100 mL) of control (C-BFV), pasteurized (P-BFV), and ultrasound-treated (UT-BFV) black figure vinegar samples. The values are expressed as the mean ± standard deviation (*n* = 3). *Indicates a statistically significant difference compared to the other groups (p < 0.05), whereas “ns” indicates significant difference.

It was observed that different processes applied to black fig vinegar had statistically significant effects on the bioactive components (Figure 4). It was determined that the total phenolic substance level in the ultrasound-treated UT-BFV group (79.77 ± 3.69 mg GAE/100 mL) was significantly higher than both the control (C-BFV) and pasteurized (P-BFV) samples (*p* < 0.05). Similarly, the UT-BFV sample had a FRAP value of 8.87 ± 0.24 mmol TE/L, which was higher than the other groups and showed a statistically significant increase, especially compared to P-BFV (*p* < 0.05) (Figure 4). An analysis of the total flavonoid content across the applied processes revealed a significant increase of approximately 20%–23% (*p* < 0.05) in the ultrasound-treated sample compared to the pasteurized sample (13.26 ± 1.12 mg CE/100 mL). On the other hand, the significant decrease observed in all bioactive compound parameters in the P-BFV sample demonstrates the adverse effect of heat treatment on these compounds. After pasteurization, the total phenolic content decreased significantly by approximately 11% compared to the control sample (66.25 ± 2.93 mg GAE/100 mL). Similarly, FRAP and TFC values were markedly lower in P-BFV compared to both C-BFV and UT-BFV (*p* < 0.05) (Figure 4). This finding aligns with the results of our study, Rguez et al. (32) reported that the application of ultrasound to onion vinegar increased total phenolic content (TPC) and total antioxidant capacity (32). Additionally, Mehrabad et al. (33) reported that applying ultrasound to apple cider vinegar increased total phenolic content, total antioxidant capacity, and total flavonoid content (33). Similarly, Shaik et al. (34) reported that ultrasound treatment applied to oat seeds resulted in significant increases in total phenolic content (TPC) and antioxidant capacity by approximately 220% and 64%, respectively, compared to the control group, by disrupting cell structure and triggering biosynthetic pathways (34). On the other hand, it has been shown that the combined application of blanching and ultrasound protects bioactive compounds by inactivating oxidative enzymes and increases the release of phenolic compounds through cavitation, thus providing higher phenolic content and antioxidant activity compared to conventional processing methods (16). Likewise, Markovinović et al. (15) the combined application of pulsed electric field (PEF) and high-power ultrasound (HPU) has been reported to enhance the extraction and stability of anthocyanins by promoting electroporation and cavitation, resulting in improved retention of bioactive compounds and greater antioxidant potential compared with conventional processing (15). These findings reveal a profile consistent with the mechanisms reported in the literature, in which ultrasound disrupts cell wall structure and facilitates the release of phenolic and flavonoid compounds from the matrix (35–39). Furthermore, Markovinović et al. (15) reported that ultrasound-treated samples exhibited a much stronger antioxidant capacity compared to untreated samples, halting cancer cell proliferation by up to 99% (18). When these results are evaluated together, it can be said that ultrasound may be a more suitable method than pasteurization for preserving and enhancing the bioactive components of black fig vinegar.

### 3.3 Phenolic profile

According to the phenolic compound profile of black fig vinegar presented in Table 5, ultrasound application (UT-BFV) results in statistically significant increases in some basic phenolics, especially compared with the control (C-BFV) and pasteurized (P-BFV) samples. For example, while caffeic acid levels were 1.39 ± 0.04 μg/mL in C-BFV and 1.09 ± 0.03 μg/mL in P-BFV, they increased to 2.94 ± 0.06 μg/mL in UT-BFV, and this increase was found to be significant as a result of variance analysis (*p* < 0.05). Similarly, while the levels of catechin hydrate were maintained at 7.50 ± 0.11 and 7.16 ± 0.05 μg/mL in the control and pasteurized samples, the value increased to 20.15 ± 1.17 μg/mL in the ultrasound process; chrysin concentrations increased from 5.23 ± 0.07 and 5.18 ± 0.04 μg/mL to 9.24 ± 0.20 μg/mL, an increase of approximately two-fold (*p* < 0.05). While the UT-BFV group exhibited higher concentrations of compounds such as hydroxycinnamic acid, vanillin, p-coumaric acid, o-coumaric acid, and trans-ferulic acid, indicating that ultrasound treatment had a significant effect on the liberation and/or rearrangement of phenolic compounds, the differences between the groups for some compounds, such as rutin, were not statistically significant (*p* > 0.05). The positive effects of ultrasound on phenolic content have also been demonstrated in various studies. In mallow vinegar, ultrasound disrupted cell walls through cavitation, increasing the release of compounds such as rutin, o-coumaric acid, vanillic acid, gallic acid, and coumarin (40). Aadil et al. (41) reported that ultrasound preserved phenolics, stilbenes, and anthocyanins in apple-grape juice, while increasing levels of chlorogenic, gallic, p-coumaric, and ferulic acids compared with pasteurization. These data suggest that ultrasound can improve product quality (41).

**TABLE 5:** Changes in total phenolic compounds of black fig vinegar samples subjected to different treatments (C-BFV, P-BFV, UT-BFV).

Phenolic compounds (μg/mL)	Samples		
	C-BFV	P-BFV	UT-BFV
**Caffeic acid**	1.39 ± 0.04^b^	1.09 ± 0.03^a^	2.94 ± 0.06^c^
**Catechin hydrate**	7.50 ± 0.11^a^	7.16 ± 0.05^a^	20.15 ± 1.17^b^
**Chrysin**	5.23 ± 0.07^a^	5.18 ± 0.04^a^	9.24 ± 0.20^b^
**Hydroxycinnamic acid**	0.34 ± 0.02^b^	0.10 ± 0.01^a^	0.30 ± 0.02^b^
**Trans-ferulic acid**	1.31 ± 0.01^b^	1.07 ± 0.02^a^	1.40 ± 0.03^c^
**Vanillin**	0.13 ± 0.01^a^	0.12 ± 0.01^a^	0.99 ± 0.04^b^
**Chlorogenic acid**	n.d.	n.d.	n.d.
**Naringenin**	1.01 ± 0.01^c^	0.77 ± 0.02^b^	0.30 ± 0.01^a^
**Resveratrol**	1.89 ± 0.11^b^	1.82 ± 0.01^b^	0.43 ± 0.01^a^
**Rosmarinic acid**	0.11 ± 0.00	0.11 ± 0.00	n.d.
**P-coumaric acid**	0.00 ± 0.00^a^	0.00 ± 0.00^a^	0.39 ± 0.02^b^
**Rutin**	13.64 ± 0.74^a^	12.50 ± 0.88^a^	13.25 ± 0.29^a^
**Salicylic acid**	0.52 ± 0.01^b^	0.52 ± 0.01^b^	0.30 ± 0.01^a^
**O-coumaric acid**	0.36 ± 0.02^a^	0.27 ± 0.04^a^	0.97 ± 0.06^b^
**Trans-cinnamic acid**	1.03 ± 0.01^c^	0.92 ± 0.02^b^	0.07 ± 0.01^a^

Control (C-BFV), Pasteurized (P-BFV), and Ultrasound-treated (UT-BFV) black fig vinegar samples; Data are presented as mean ± standard deviation (*n* = 3); n.d.: could not be detected. The presence of varying superscript letters indicates statistically significant differences between treatments (*p* < 0.05; one-way ANOVA followed by Tukey’s test).

However, Table 5 also shows that ultrasound did not yield a unidirectional increase in all phenolics. Naringenin levels were reported as 1.01 ± 0.01, 0.77 ± 0.02, and 0.30 ± 0.01 μg/mL in the control and pasteurized samples, respectively. Ultrasound-treated samples, respectively, and as 1.89 ± 0.11, 1.82 ± 0.01, and 0.43 ± 0.01 μg/mL for resveratrol, with the decrease observed in the ultrasound group for both compounds being statistically significant (*p* < 0.05). Similarly, the values decreasing from 0.52 ± 0.01 to 0.30 ± 0.01 μg/mL in salicylic acid and from 1.03 ± 0.01 to 0.07 ± 0.01 μg/mL in trans-cinnamic acid suggest that ultrasound-induced cavitative effects may cause losses in some phenolics through oxidation or degradation. The lack of detection of chlorogenic acid across all groups and of rosmarinic acid in the ultrasound group (n.d.) highlights the critical role of the interaction among matrix properties, processing conditions, and compound stability. This holistic perspective suggests that ultrasound parameters should be optimized not only to increase total phenolic levels but also to allow for statistically significant shaping of the desired specific phenolic profile. Erdal et al. (42), in line with our study, reported that ultrasound treatment in gilaburu vinegar increased the level of phenolics such as ferulic acid, vanillic acid, rutin, o-coumaric acid, and p-coumaric acid, which also contributed to the optimization of the process by providing significant changes in anthocyanins and flavanols (42).

### 3.4 Bioaccessibility and digestive stability

The results presented in Table 6 show that there is a gradual and significant decrease in TPC, TFC, and FRAP values in black fig vinegar samples as they pass from the non-digestive phase to the intestinal phase. When evaluated in terms of TPC, the ultrasound-applied UT-BFV sample had a value of 79.77 3.69 mg GAE/100 mL in the non-digested phase, whereas the C-BFV and P-BFV samples had levels of 74.25 ± 1.57 and 66.25 ± 2.93 mg GAE/100 mL, respectively. The statistical difference indicated by superscript letters suggests that the difference between UT-BFV and P-BFV is particularly significant (*p* < 0.05). When the oral phase results are examined, it is observed that TPC decreased to 63.82 ± 2.95 mg GAE/100 mL in UT-BFV and to 52.90 ± 2.50 mg GAE/100 mL in P-BFV. A similar trend was observed for the FRAP parameter, with the UT-BFV sample maintaining a level of 6.83 ± 0.18 mmol TE/L, while the P-BFV remained at 5.56 ± 0.18 mmol TE/L. These findings show that although the reduction due to digestion was observed in all samples, the ultrasound process contributed to the preservation of phenolic compounds and maintained a higher reducing capacity. In a study by Tokatli Demirok (40), comparing different pasteurization processes applied to Valencia orange juice with the ultrasound treatment, it was reported that the least component loss occurred in the ultrasound-treated samples during *in vitro* digestion, and these samples had significantly higher digestive stability. These results indicate that ultrasound treatment can better protect the bioactive components in orange juice against degradative effects, such as superoxide dismutase degradation, during ingestion, compared with pasteurization (39). Similarly, a study conducted by Levent et al. (31) on garden cress juice reported that ultrasound significantly reduced the decrease in TPC and FRAP values throughout the digestive phases compared to pasteurized samples. The study noted that this protective effect was highest in the oral and gastric phases after ultrasound compared to pasteurization (26).

**TABLE 6:** Changes in FRAP, TFC, and TPC as a function of simulated gastrointestinal digestion of black fig vinegar samples under various treatment conditions.

Phases	Samples	TPC (mg GAE/100 mL)	FRAP (mmol TE/L)	TFC (mg CE/100 mL)
**Undigested**	C-BFV	74.25 ± 1.57^ab^	8.12 ± 0.75^a^	11.65 ± 0.85^a^
	P-BFV	66.25 ± 2.93^a^	7.27 ± 0.17^a^	10.77 ± 0.91^a^
	UT-BFV	79.77 ± 3.69^b^	8.87 ± 0.24^a^	13.26 ± 1.12^a^
**Oral digestion**	C-BFV	59.40 ± 1.26^ab^	6.08 ± 0.38^ab^	9.09 ± 0.66^a^
	P-BFV	52.90 ± 2.50^a^	5.56 ± 0.18^ab^	8.40 ± 0.71^a^
	UT-BFV	63.82 ± 2.95^b^	6.83 ± 0.18^b^	9.96 ± 0.32^a^
**Gastric digestion**	C-BFV	38.02 ± 0.80^ab^	4.07 ± 0.37^a^	5.82 ± 0.42^a^
	P-BFV	33.93 ± 1.48^a^	3.66 ± 0.11^a^	5.39 ± 0.46^a^
	UT-BFV	40.85 ± 1.89^b^	4.44 ± 0.12^a^	6.62 ± 0.55^a^
**Intestinal digestion**	C-BFV	23.56 ± 0.49^ab^	2.47 ± 0.15^ab^	3.61 ± 0.26^a^
	P-BFV	21.04 ± 0.92^a^	2.26 ± 0.05^a^	3.31 ± 0.32^a^
	UT-BFV	25.40 ± 1.06^b^	2.78 ± 0.05^b^	4.00 ± 0.25^a^
**Recovery %**	C-BFV	31.72 ± 0.01^a^	30.40 ± 0.98^a^	30.95 ± 0.01^a^
	P-BFV	31.76 ± 0.01^a^	31.02 ± 0.04^a^	30.67 ± 0.38^a^
	UT-BFV	31.85 ± 0.15^a^	31.29 ± 0.29^a^	30.16 ± 0.67^a^

C-BFV, control black fig vinegar; P-BFV, thermal pasteurized black fig vinegar; UT-BFV, ultrasound-treated black fig vinegar; Values are expressed as mean ± standard deviation (*n* = 3). mg GAE, milligram gallic acid equivalent; FRAP, ferric reducing antioxidant power; TPC, total phenolic content. Significant disparities (*p* < 0.05) between treatments were indicated by the utilization of different superscript letters within the same digestion phase.

The gastric and intestinal phase results also support a similar trend. TPC values were determined as 40.85 ± 1.89 mg GAE/100 mL in the gastric phase and 25.40 ± 1.06 mg GAE/100 mL in the intestinal phase in UT-BFV. In P-BFV, TPC decreased to 33.93 ± 1.48, 21.04 ± 0.92 mg GAE/100 mL in the same phases. FRAP results also confirmed this difference, showing that in the intestinal phase, UT-BFV had a value of 2.78 ± 0.05 mmol TE/L, while P-BFV remained at 2.26 ± 0.05 mmol TE/L (*p* < 0.05). However, when the recovery percentages after digestion were evaluated, they ranged from 31.7% to 31.9% for TPC, 30.4%–31.3% for FRAP, and 30.2%–31.0% for TFC, and there was no significant difference between the treatment groups. This suggests that the different treatments applied affect the initial levels of phenolic compounds and the losses during digestion, but do not significantly change the proportional distribution of the bioaccessible fraction after digestion. Therefore, ultrasound, pasteurization, and control processes create distinct effects that should be carefully compared in terms of the preservation of phenolic stability and antioxidant capacity during digestive studies on fruit juices, Yıkmış et al. (28) and Wang et al. (43) reported that ultrasound application significantly reduces the loss of bioactive components compared to pasteurization, thereby better preserving their bioavailability (43, 44). In a separate study on jujube vinegar, ultrasound treatment was reported to increase phenolic compounds, flavonoids, and antioxidant capacity compared with the conventional pasteurization method. These findings suggest that technologies, such as ultrasound, offer significant potential to enhance the functional value of food products (28). However, further research on bioavailability is needed to evaluate and compare losses during digestion fully.

### 3.5 Correlation and PCA of phenolics and antioxidant capacity

**FIGURE 5:**
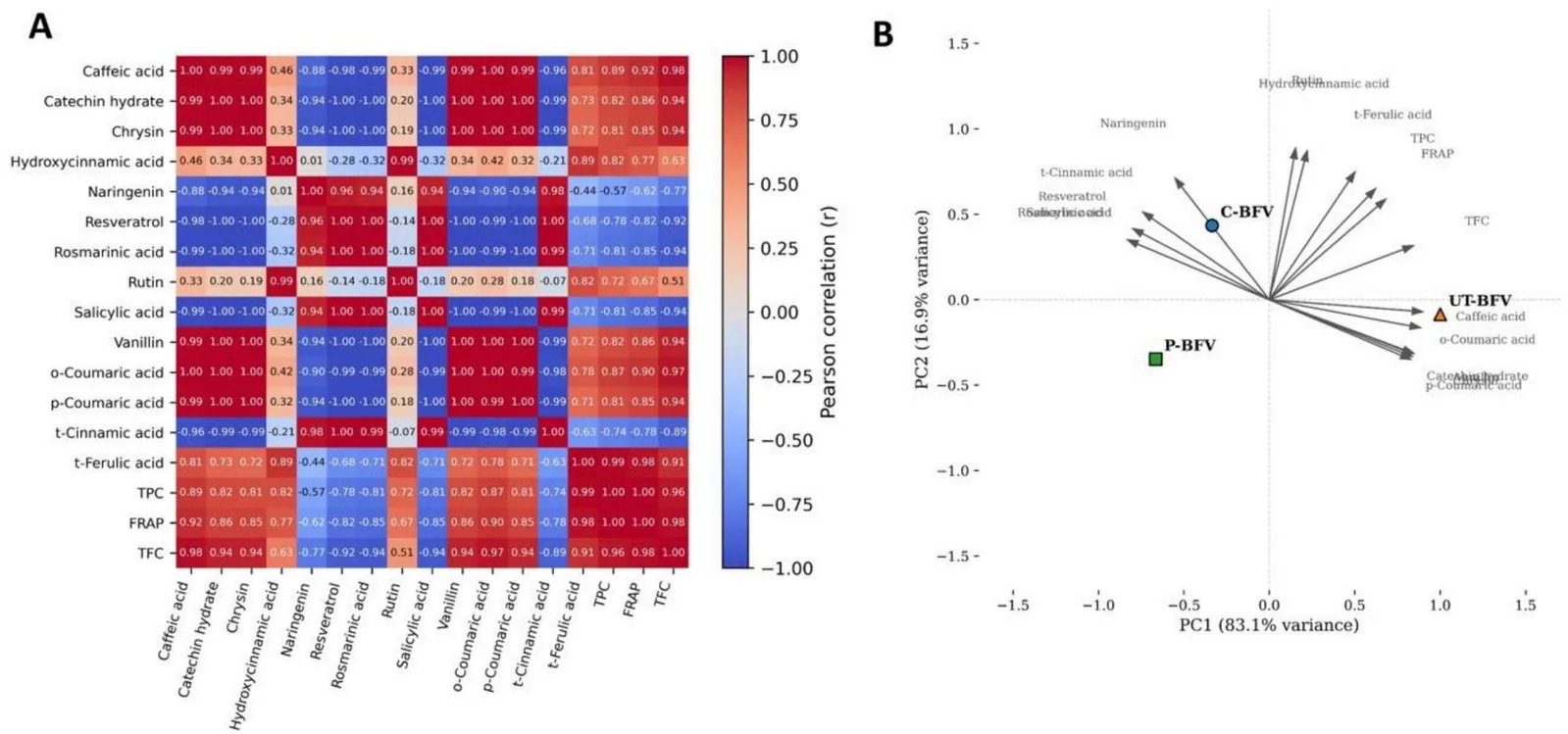
**(A)** Pearson correlation heatmap showing the relationships between individual TPC (total phenolic content), TFC (Total flavonoid content), and FRAP (ferric reducing antioxidant power) values in black figure vinegar. **(B)** Principal component analysis (PCA) biplot (PC1, 83.1% variance; PC2, 16.9% variance) illustrating the distribution of control (C-BFV), pasteurized (P-BFV), and ultrasound-treated (UT-BFV) black figure vinegar samples in relation to phenolic compounds and antioxidant indices.

The Pearson correlation heat map in Figure 5A shows powerful, statistically significant relationships between the phenolic compounds in black fig vinegar and TPC, TFC, and FRAP values. In particular, most of the correlation coefficients between o-coumaric acid, caffeic acid, trans-ferulic acid, rutin, and rosmarinic acid, and TPC, TFC, and FRAP are observed at r ≥ 0.90 and *p* < 0.05, demonstrating that these phenolics are the “major contributing determinants” to the total antioxidant capacity of the product. Furthermore, the high positive correlations between phenolic compounds (mainly in the *r* = 0.80–0.99 range) suggest that they respond to the same biochemical pathways and similar processing conditions, indicating that it would be more rational to consider these compounds as a holistic phenolic network structure rather than individually for process optimization.

The PCA biplot (PC1: 83.1%; PC2: 16.9%) presented in Figure 5B holistically visualizes the distinction between control (C-BFV), ultrasound-treated (UT-BFV), and pasteurized (P-BFV) black fig vinegar samples, as well as the relationship between the phenolic profile and antioxidant parameters. The positioning of the UT-BFV sample closer to the caffeic acid and o-coumaric acid vectors, as well as the TPC/TFC/FRAP arrows, suggests that ultrasound treatment increases antioxidant capacity through the preservation and/or liberation of phenolic compounds. In contrast, the clustering of the P-BFV sample in a different region indicates that heat treatment may limit both the phenolic profile and antioxidant properties. At the same time, the intermediate position of the C-BFV suggests that the conventional matrix can be preserved, with non-thermal processes strategically used for targeted phenolic enrichment.

## 4 Conclusion

The present study demonstrated that ultrasound processing is a promising non-thermal alternative to conventional pasteurization for improving the functional quality of black fig vinegar. Under the optimized processing conditions determined by Response Surface Methodology, ultrasound treatment significantly enhanced total phenolic content, total flavonoid content, FRAP values, and the concentrations of several individual phenolic compounds compared with both untreated and pasteurized samples. These improvements are likely associated with ultrasound-induced cavitation, which promotes cell wall disruption and facilitates the release of bound phenolic compounds while minimizing the thermal degradation commonly observed during conventional pasteurization. The integration of Response Surface Methodology with data augmentation and ensemble machine learning successfully validated the optimized processing conditions. Among the evaluated algorithms, the Extra Trees model provided the highest predictive performance, accurately estimating TPC and FRAP values, demonstrating the potential of artificial intelligence as a reliable tool for optimizing non-thermal food processing. Although simulated gastrointestinal digestion resulted in reductions in phenolic compounds and antioxidant capacity in all treatments, ultrasound-treated samples consistently retained higher absolute levels of these bioactive compounds than pasteurized and untreated vinegar. These findings indicate that ultrasound processing not only enhances the initial functional quality of black fig vinegar but also improves the retention of bioactive compounds during digestion, thereby increasing its potential as a functional food. Overall, this study provides a novel framework by combining ultrasound optimization, Response Surface Methodology, data augmentation, ensemble machine learning, phenolic profiling, and *in vitro* bioaccessibility assessment within a single approach. This integrated strategy offers a sustainable and efficient methodology for developing high-value functional fermented foods while reducing the adverse effects associated with conventional thermal processing. A limitation of the present study is that bioaccessibility was evaluated using an *in vitro* gastrointestinal digestion model, which cannot fully represent the complexity of human digestion and metabolism. Therefore, future studies should validate these findings through *in vivo* experiments and clinical investigations. Furthermore, the proposed machine-learning-assisted optimization strategy should be extended to different fruit vinegars and other fermented food matrices to evaluate its robustness and industrial applicability. Additional research focusing on storage stability, sensory quality, and pilot-scale or industrial-scale ultrasound processing would further facilitate the commercialization of ultrasound-assisted functional vinegar production.

## Data Availability

The raw data supporting the conclusions of this article will be made available by the authors, without undue reservation.
